# Improving hospice delirium guideline adoption through an understanding of barriers and facilitators: A mixed-methods study

**DOI:** 10.1371/journal.pone.0310704

**Published:** 2024-09-26

**Authors:** Catriona Jackson, Catherine Malia, Hannah Zacharias, Judith Dyson, Miriam J. Johnson

**Affiliations:** 1 Wolfson Palliative Care Research Centre, Hull York Medical School, Hull, East Yorkshire, United Kingdom; 2 Leeds Teaching Hospitals NHS Trust, Leeds, West Yorkshire, United Kingdom; 3 St Gemma’s Hospice, Leeds, West Yorkshire, United Kingdom; 4 Centre for Social, Health and Related Research, Birmingham City University, Birmingham, West Midlands, United Kingdom; Global Health Neurology Lab / NSW Brain Clot Bank, NSW Health Pathology / Liverpool Hospital and South West Sydney Local Health District / Neurovascular Imaging Lab, Clinical Sciences Stream, Ingham Institute, AUSTRALIA

## Abstract

**Objectives:**

This study seeks to understand and address barriers to practitioners’ optimal assessment and management of people with delirium in hospices.

**Methods:**

Retrospective clinical record review to identify areas of low concordance with guideline-adherent delirium care; Survey of healthcare practitioners to identify barriers and facilitators to optimal care; Qualitative interviews with health care practitioners to explore and develop strategies to address barriers or optimise facilitators; Meeting with senior clinical staff to refine identified strategies.

**Results:**

Eighty clinical records were reviewed. Elements of poor guideline concordance were identified. Delirium screening on admission was conducted for 61% of admissions. Non-pharmacological management was documented for 59% of those we identified as having delirium from the clinical records. Survey and interview data identified key barriers to delirium assessment as competing priorities, poor knowledge and skills and lack of environmental resources (staff and guidelines, environment). Consultation with staff resulted in strategies to address barriers and enhance facilitators including champions, educational meetings, audit and feedback, and environmental changes (including careful consideration of the staff skills mix on shift and tools to support non-pharmacological management).

**Conclusions:**

We conducted a theoretically underpinned, internationally relevant study in a hospice in England, UK. Implementation of strategies should result in greater guideline-adherent delirium care. Further work should test this in practice and include both process and clinical outcomes (e.g., reduction in delirium days).

## Introduction

Delirium is characterised by acute onset of altered awareness and attention with impaired cognition [1]. Patients receiving specialist palliative care are at high risk of delirium [1, 2]; 60% of patients will have delirium during a hospice admission [3]. Delirium is associated with prolonged hospitalisation, increased mortality [4], higher care costs [5] and distress for patient, family and carers [6].

Best care guidelines focus on prevention, recognition and management [7, 8]. *Prevention* includes measures such as orientation (e.g. signage, familiar visitors), optimising sleep, maintaining mobility, nutrition, hydration, bladder and bowel function and managing pain and medication [7, 8]. *Recognition* is advised using screening tools [7, 8]. The 4AT rapid clinical test is brief, easy to use without training and performs well in terms of sensitivity [8]. *Management* involves identification and treatment of reversible causes and non-pharmacological care using same strategies as for prevention alongside assessment and management of distress, and prevention of complications (e.g. falls, pressure ulcers) [7, 8]. Pharmacological management is recommended only when the patient doesn’t respond to other measures and is severely distressed, or a risk to themselves or others [7, 8]. Delirium is thought to be reversible in 27% of palliative care patients [9]. Concordance with optimal delirium care is variable e.g. only 37% of hospice staff use a screening tool to recognise delirium [10].

**Fig 1:**
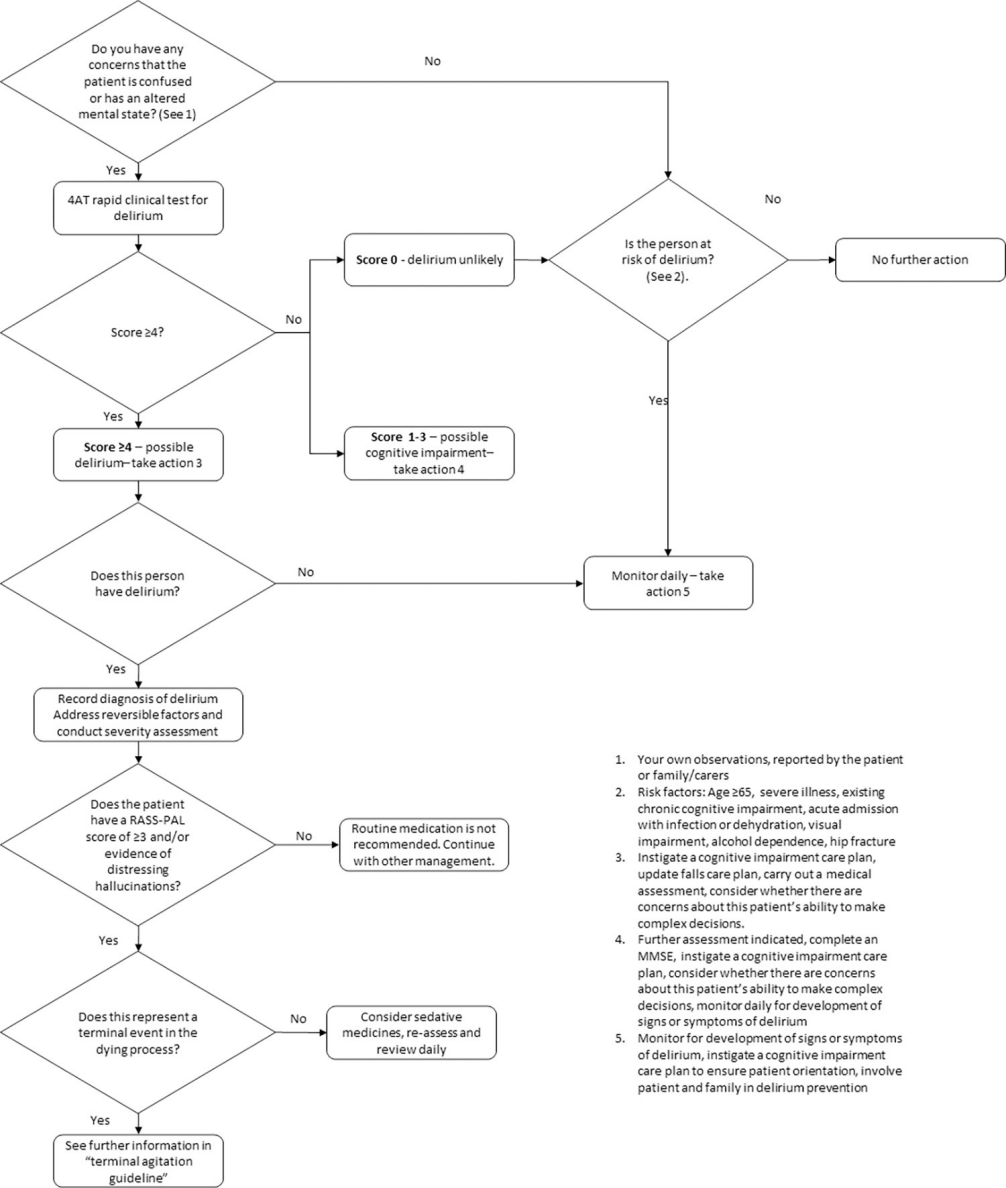
The optimised process of delirium care featured in the hospice delirium guideline, post initial quality improvement project.

This study was conducted at a hospice in England, building on an earlier quality improvement project (QIP) (August 2016 –July 2019). Through audit, QIP showed delirium was under-recognised and reversible causes were rarely sought. Sequential guideline changes alongside mandatory training in their use, standardised supporting documentation including a delirium screening tool and care plan, and delirium champions were introduced. The QIP optimised guideline introduced the 4AT [8] and Richmond Agitation-Sedation Scale modified for palliative care inpatients (RASS-PAL) [11] (Fig 1). Initial improvements in care occurred but were not sustained. Failure to deliver sustained change, despite standard quality improvement approaches, was the reason for this study.

The study reported was conducted between August 2019 and July 2021 and included i) Clinical record review to identify areas of low concordance with guideline-adherent delirium care; ii) a survey of healthcare practitioners to identify barriers and facilitators to optimal care; iii) qualitative interviews with healthcare practitioners to explore and develop strategies to address barriers or optimise facilitators; and iv) a meeting with senior clinical staff to refine identified strategies. In this paper we aimed to i) identify areas of poor-concordance with delirium guidelines, ii) identify barriers and facilitators to concordance and iii) design strategies to improve delirium guideline adherence.

## Methods

### Design

The theoretical underpinning of this quality improvement project study was the Theoretical Domains Framework (TDF) [12, 13] and Normalisation Process Theory (NPT) [14].

Adopting a practice guideline requires behavioural change [15]; the TDF is a framework consisting of all published models of behaviour or behaviour change and therefore includes all potential modifiable determinants. This supports assessment of barriers and facilitators and guides selection of behaviour change techniques likeliest to be effective [13]. NPT considers incorporating complex interventions into everyday practice within the wider context of organisational structures, social norms, and group processes [14].

French et al. describe a four-step systematic approach to applying the TDF to develop a theory-informed implementation intervention [16]. This study is structured using the first three steps.

### Setting

The study took place at one hospice in England: an independent charity receiving some NHS funding and providing specialist palliative care including in-patient and community-based.

### Step 1: Who needs to do what differently?

#### Clinical record review: target behaviours

*Purpose*. To establish concordance with expected clinical behaviours relating to prevention, recognition, and management of delirium (Fig 1). To identify key ‘target behaviours’ for inclusion in the survey.

*Participants.* A retrospective clinical record review of 80 consecutive in-patient records.

*Data collection*. Clinical record data were extracted in August 2019 (by CJ) and recorded using a *pro forma* designed to capture demographic information and elements of delirium care. This *pro forma* had been used in the previous QIP. A validated tool was used to retrospectively identify delirium from the clinical record [17]. Where data were ambiguous, cases were discussed (with MJ) and a joint decision recorded.

*Analysis.* Findings are presented using descriptive statistics (frequencies and percentages).

The two behaviours least adhered to, or judged as key in enabling downstream behaviours, were selected as ‘target behaviours’ for inclusion the survey. Target behaviours were defined using the AACTT (Action, Actor, Context, Target and Time) framework [18].

### Step 2: Using a theoretical framework, which barriers and facilitators need to be addressed?

#### Staff survey

*Purpose*. To establish embeddedness (normalisation) of the delirium guideline using items from the normalisation measure development (NoMAD) instrument [19] (based on NPT) and establish barriers and facilitators to adoption of guidelines (based on the TDF). Embeddedness refers to whether, and to what extent, process have occurred to allow the carrying out of delirium care procedures to become a routine part of the work of staff in the hospice.

*Participants*. All (n = 76) doctors, nurses, healthcare assistants and pharmacists at the hospice involved in delivering in-patient care to patients with delirium were eligible to participate.

*Identification*, *consent and data collection*. All eligible staff were emailed with survey information and a link to an electronic version on the Qualtrics (XM) platform. Paper surveys were available in envelopes on hospice wards and in the staff canteen and completed surveys sealed and returned to the project team in person, through internal mail or ward managers. Responses were anonymous. Survey completion was taken as implied consent.

The survey was developed based on the NOMAD instrument; a validated instrument designed utilising NPT. The customisable NOMAD questions were adapted to reflect the nature of this project. NOMAD instrument questions were mapped to the behaviour change Theory theoretical domain framework (TDF) domains to determine which NOMAD instrument questions already covered aspects of the TDF. This allowed identification of domains not covered by the NOMAD instrument. Additional questions were added to the survey to cover the missing TDF behaviour domains.

The survey included questions relating to i) demographic information to establish a representative sample and differences between practitioner groups and ii) five-point Likert scale items with points ranging from strongly agree to strongly disagree. Participants were asked to include contact details if they were willing to take part in interviews.

*Analysis*. Findings are presented using descriptive statistics (frequencies and percentages).

### Step 3: Which intervention components could overcome the modifiable barriers and enhance facilitators?

#### Staff interviews

*Purpose*. To obtain further details regarding identified barriers and facilitators to delirium guideline adoption and strategies to address or enhance these.

*Participants*. Inclusion criteria were as for staff survey. We sought to recruit 15 interview participants to achieve data saturation [20].

*Sampling*. In addition to the invitation to interview at the end of the survey, purposive sampling staff took place through emails with information and instruction to contact the researcher directly if interested in participating.

*Data collection*. Semi-structured interviews were all conducted by CJ, a female, clinician and researcher, familiar with the hospice and with some prior professional contact with participants. A topic guide was used; questions related to barriers and facilitators identified and strategies to address or enhance these. Most were face-to-face at the hospice, but some opted for telephone interview due to COVID-19. Written or verbal consent was given prior to interviews, including for audio-recording and transcription.

*Analysis*. Analysis was conducted in three stages. Data relating to barriers, facilitators and solutions were subjected to framework analysis [21]. Line-by-line coding of the first two transcripts was independently completed (CJ and MJ). The coding was done both deductively using behaviour change theory theoretical domains and behaviour change techniques and inductively reflecting other ideas found in the transcripts. Analysis proceeded in parallel with collection until saturation was achieved. This allowed identification of barriers, facilitators and strategies to address them.

Barriers and facilitators were prioritised by identifying which were of high importance using published criteria: i) frequency of reporting and ii) importance to participants [22]. These were selected to be addressed or enhanced in practice.

The prioritised barriers and facilitators were categorised to behaviour change theoretical domain framework (TDF) domains. The behaviour change techniques (BCTs) that have been linked to those TDF domains were then identified using the mapping matrix as established in an expert consensus process by Michie et al [12]. BCTs are the active components of an intervention and either encourage the desired behaviour or deter an undesired behaviour. In this intervention, strategies identified in the interviews that reflected BCTs were utilised, the choice was guided by a chartered psychologist (JD).

NPT advocates that people involved in carrying out an intervention need to continue to invest in the intervention in all four core construct areas to ensure it becomes embedded and integrated in practice. To provide the best chance of integration and embedding of the intervention, we took the planned intervention components (BCTs) and analysed these alongside the least fulfilled NPT core/sub constructs (staff survey)–the areas which were least embedded. Where there was strong alignment between an NPT construct and a BCT we designed an implementation strategy for that BCT that addressed the weakness in current embeddedness.

#### Staff meeting

*Purpose*. To determine feasibility of planned strategies to enhance adoption of delirium guidelines and consider modifications.

*Participants and invitation*. Senior management staff (medical and nursing) were invited by email to attend a presentation and discussion of barriers, facilitators and suggested strategies to enhance adoption of delirium guidelines.

*Data collection*. The barriers, facilitators and suggested strategies identified by survey and interview data were presented by CJ. Participants were asked to consider them according to the criteria of the APEASE (Acceptability, Practicability, Effectiveness and cost-effectiveness, Affordability, Safety and Equity) framework [18]. Field notes were taken.

*Analysis*. Data from field notes were used pragmatically to modify suggested solutions.

## Results

### Step 1—who needs to do what differently?

#### Clinical record review

The clinical records of 80 in-patient episodes were reviewed, 47.5% (n = 38) were men, mean age was 73.6 (range 34 to 97), median length of stay was 7.5 days (range 0 to 75) and 82.5% (n = 66) of patients had cancer. Analysis identified 44 episodes of delirium, half on admission and half developed during hospice stay. Table 1 illustrates adherence with the delirium guideline clinical behaviours.

**Table 1:** Concordance with delirium guidelines.

Expected clinical behaviour for all patients		Concordance observed/ relevant (%)
**Prevention**	Risk assessment conducted for patients on admission (eligible if not screened for delirium, or had a negative delirium screen)	38/65 (58)
	Delirium prevention measures undertaken in patients with a positive risk screen	5/34 (15)
**Recognition**	Screening on admission	49/80 (61)
	Medical assessment for those with evidence of confusion or positive delirium screen on admission	12/30 (40)
	Episodes of confusion during admission screened for delirium	1/32 (3)
	Medical assessment for episodes of confusion during admission (eligible if had a positive delirium screen, or a new confusion that wasn’t screened for delirium)	9/32 (28)
	Episodes of delirium diagnosed by staff	17/44 (39)
**Expected clinical behaviour for those with episodes of delirium**		**Number (n = 44) (%)**
**Non-pharmacological management**	Delirium care plan	24 (54)
	Appropriate non-pharmacological management	26 (59)
	Systematic assessment of reversible causes (more than one reversible cause of delirium explicitly considered as a cause of delirium)	23 (52)
**Pharmacological management**	Symptom severity assessment to guide pharmacological management	13 (29)
	• *Hallucination assessment alone*	*4 (9)*
	• *RASS-PAL alone*	*0*
	• *RASS-PAL and Hallucination Assessment*	*9 (20)*
	Pharmacological management as per guideline^a^	32 (73)

^a^ The guideline relating to pharmacological management for delirium was followed: (i) If the patient **did not meet** the criteria to receive pharmacological management then they did not receive pharmacological management. (ii) If the patient **did meet** criteria for pharmacological then they received pharmacological management using medication as outlined in the delirium guideline.

Two clinical behaviours were selected for inclusion in the survey. The first step in delirium guideline process (Fig 1), *screening on admission*, had only 60% concordance AND is an enabling factor for all subsequent, downstream delirium guideline components. The second area of low concordance (according to documentation) was *non-pharmacological management*. The behaviours were defined as:

- *‘The admitting clinician (doctor*, *nurse or healthcare assistant)* (actor) *to screen* (action) *the patient* (target) *for delirium on admission* (time) *to the hospice* (context)*’ and*
- *‘The clinician (nurse or healthcare assistant)* (actor) *to deliver non-pharmacological management strategies (e*.*g*. *display clock in patient room*, *ensure patient has hearing aids and glasses)* (action) *subsequent to a patient* (target) *being assessed to have delirium* (time) *in the hospice* (context)*’*.

### Step 2—using a theoretical framework, which barriers and facilitators need to be addressed?

#### Survey

**Fig 2:**
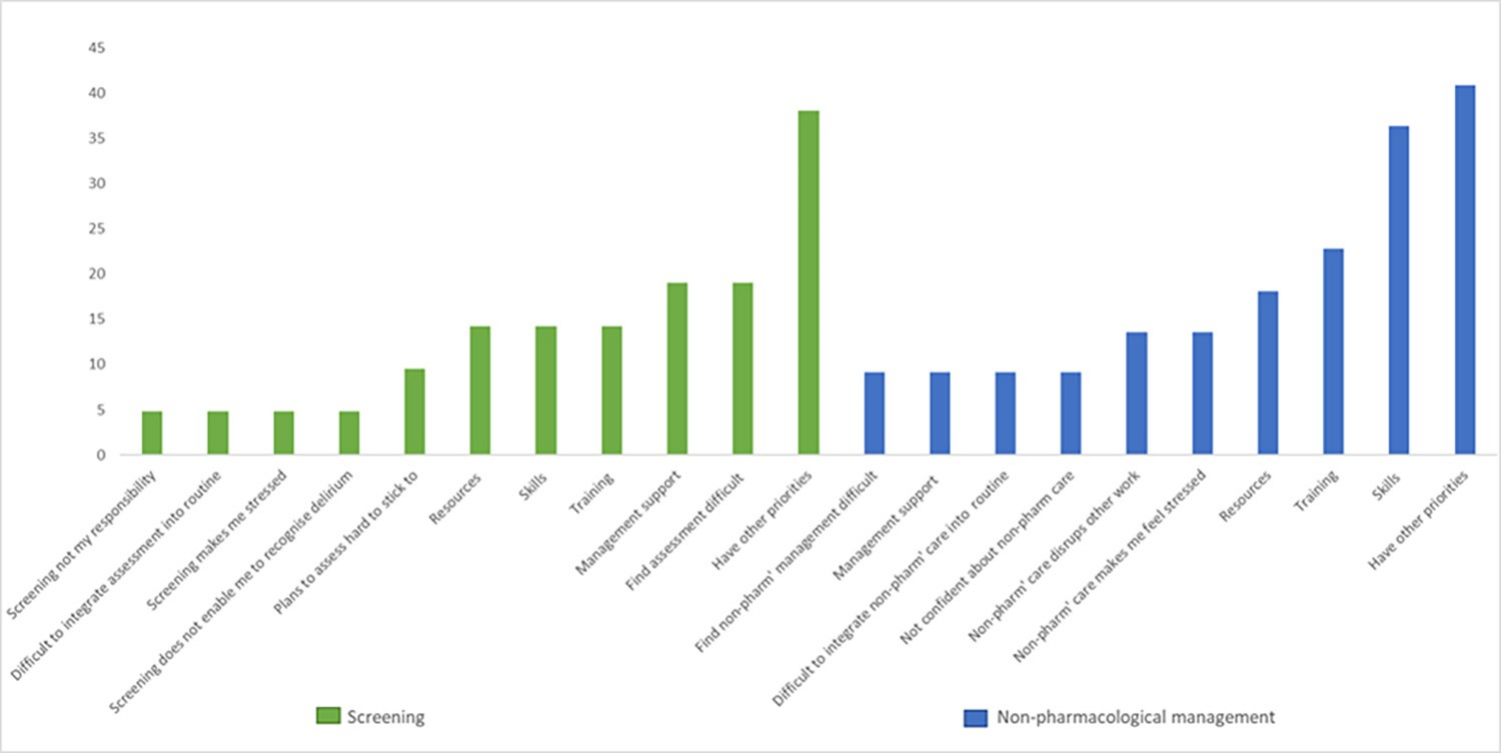
Barriers to target behaviours (%).

Surveys were completed by 26/76 (34%) possible participants. Table 2 illustrates participant characteristics (representative of the hospice staffing profile). Fig 2 demonstrates the items where respondents indicated barriers to either target behaviour. All identified barriers were explored further in interviews and specific solutions to barriers sought.

**Table 2:** Survey participant characteristics.

Characteristics		Number
**Time worked for the hospice** (years)	<2	11
	2–5	6
	6–10	2
	11–15	3
	>15	4
**Job category**	Medical consultant	2
	Junior doctor (below consultant grade)	4
	Senior registered nurse (nurse practitioner or ward sister)	3
	Junior registered nurse or student nurse	8
	Healthcare assistant	8
	Allied health professional	1
**Role in relation to the guideline** (staff could have more than one role)	Involved in developing, managing, or overseeing	3
	Involved in delivering patient care according to the guideline	24

Table 3 shows the median responses to the Likert item questions mapping to NPT constructs. This provides a measure of which constructs scored most positively and negatively.

**Table 3:** Survey results summary of normalisation process theory Likert item question responses.

Normalisation Process Theory construct		Questions	Median response (IQR) 1 = most strongly negative score 5 = most strongly positive
**Coherence**		Staff in this organisation have a shared understanding of the purpose of the delirium guideline	4 (4–4.25)
		I can see the potential value of the delirium guideline for my work	
**Cognitive Participation**		There are key people who drive the delirium guideline forward and get others involved	4 (4–5)
		I’m open to working with colleagues in new ways to use the delirium guideline	
		I believe that participating in the delirium guideline is a legitimate part of my role	
		I will continue to support the delirium guideline	
**Collective action**	**Overall**		4 (3–4)
	**Interactional workability**	I can easily integrate delirium screening into my existing work	4 (4–4)
		I can easily integrate non-pharmacological management of delirium into my existing work	
	**Relational integration**	Delirium screening disrupts working relationships	4 (3–4)
		I have confidence in other people’s ability to use delirium screening	
		Non-pharmacological management of delirium disrupts working relationships	
		I have confidence in other people’s ability to use non-pharmacological management of delirium	
	**Skill set workability**	Work is assigned to those with skills appropriate to delirium screening	4 (3–4)
		Sufficient training is provided to enable staff to implement delirium screening	
		Work is assigned to those with skills appropriate to non-pharmacological management of delirium	
		Sufficient training is provided to enable staff to implement non-pharmacological management of delirium	
	**Contextual integration**	Sufficient resources are available to support delirium screening	4 (3–4)
		Management adequately supports delirium screening	
		Sufficient resources are available to support non-pharmacological management of delirium	
		Management adequately supports non-pharmacological management of delirium	
**Reflexive monitoring**		I am aware of reports about the effects of the delirium guideline	4 (3–4)
		The staff agree that the delirium guideline is worthwhile	
		I value the effects that the delirium guideline has on my work	
		I can modify how I work with the delirium guideline	

### Step 3—which intervention components could overcome the modifiable barriers and enhance facilitators?

#### Interviews

**Fig 3:**
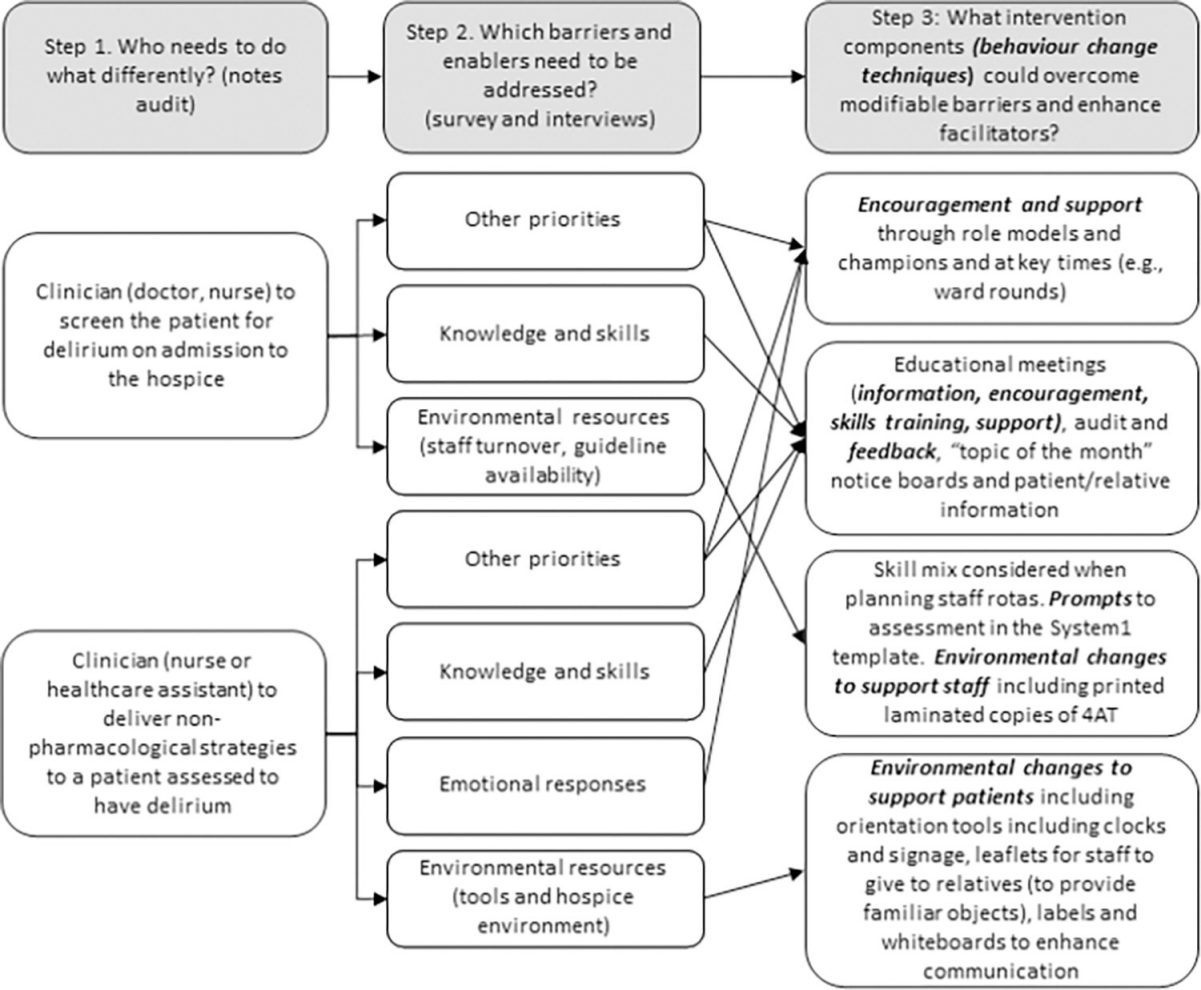
Intervention development process.

Eight Interviews were conducted averaging 28 minutes. Participants were one senior doctor (senior registrar or over), three senior nurses (nurse practitioner or ward sister), one junior nurse, two junior doctors and one healthcare assistant (HCA). Due to the small number of participants, to maintain anonymity when offering direct quotations, we have identified participants as doctors (all grades) or nurses (all grades and including the HCA). There were three categories of barriers for screening and four for non-pharmacological management. Each are presented below followed by a summary of strategies suggested at interview, and modifications proposed in the meeting. A summary is presented in Fig 3.

#### Barriers and facilitators to delirium screening

*Other priorities*. Within the wider context of hospice work, participants cited many competing priorities: ‘*there’s a big push at the moment*… *we’ve got a CQC [Care Quality Commission] inspection coming up’* (Participant (P) 5, nurse (N)). Collecting other (non-delirium) information was prioritised: ‘*does naturally drop down my priority list compared to*, *say*, *advanced care planning or escalation decisions’* (P4, doctor (D)).

*Knowledge and skills*. Some participants, often more junior staff, seemed unclear what delirium was. Participants used ambiguous terminology such as agitation, confusion or distress, or conflated delirium with terminal agitation or Alzheimer’s disease. Participants considered recognition of hypoactive delirium particularly difficult. Some participants were unaware of the correct screening test and two cited tests that do not screen for delirium: ‘*To*, *like*, *diagnose a delirium we would probably look to*… *doing a mini-mental’* (P2N). Many, especially nurses, seemed to rely on a ‘sense’ something was wrong as a way of recognising delirium.

*Environmental resources*. Poor staffing levels, skill mix and unavailable guidelines were barriers to recognising delirium; *‘doctors now rotate*… *at a junior level there are no permanent members of staff here’* (P4D). Day to day changes in staffing level was cited as a reason recognition of delirium might vary. The perceived availability of guidelines varied, some found the online portal inaccessible, others said paper copies were not provided.

#### Barriers and facilitators to non-pharmacological management

*Other priorities*. Many participants noted other care was prioritised over non-pharmacological management of delirium: *‘They’re so*… *‘right I’m gonna get them washed*, *and we’ll give them their tablets and…’* (P3N). Others suggested less drive to provide care to hypo-actively delirious patients as they are perceived as easy to manage: ‘*A patient*… *flat affect and sleeping a lot of the time doesn’t create a lot of issues ostensibly’* (P1D).

*Knowledge and skills*. Knowledge or skills barriers for non-pharmacological treatment were frequently cited. Examples of each included: *‘I hear people saying*, *“what use is turning the light off*?*”*… *or whatever*… *when the patient is crawling the walls’* (P3N).

*Emotional responses*. Several participants spoke of the distress they felt when seeing a patient with delirium. This led in some cases to an inappropriate management. One participant described frustrations of managing people with delirium as barriers to care: *‘Sometimes we see people walking off and think “Oh*, *for God’s sake*…*”‘* (P8N). Participants suggested leaning toward pharmacology to protect families from distress.

*Environmental resources*. Environmental barriers and facilitators included available tools and the ward structure. Some thought there were lots of tools to assist, some considered useful, others less so: *‘The pressure mats*… *but often a delirious patient thinks it’s a hole in the floor’* (P3N). Others said some tools were not accessible or used or well maintained. The ward set-up could be challenging: *‘the [dividing] curtain*… *blocks out the light*… *she thinks it’s the night-time’* (P6D). However, most patients were in private rooms, which was considered facilitative.

#### Suggested strategies

To address ***competing priorities*** for both screening and non-pharmacological management of delirium, and to support **emotional barriers,** participants suggested ***role modelling*** and ***champions*.** This included discussions in handover, a practice educator, and junior staff observing senior colleagues: *‘A healthcare assistant sits in on the admission’* (P8N). ***Educational meetings*** were suggested to address ***knowledge and skills*** barriers. Some suggested these should be mandatory and regular to ensure attendance and keep the subject current. ***Audit and feedback*** were popular suggestions across all elements of the guideline. Other suggestions included a ‘***topic of the month’ notice board*** *and* ***patient and relative information leaflets*** as a roundabout way of providing information to staff. To address ***environmental*** barriers to screening, participants suggested careful consideration of ***skill mix*** on shift. To improve access to guidelines, participants suggested ***prompts*** on the electronic record and ***printed guidelines and screening tools*.** In response to ***environmental*** barriers to non-pharmacological treatment, strategies included the provision of ***orientation tools*** to all patients irrespective of whether they had symptoms of delirium, and some participants suggested families bring in ***familiar objects***.

*Implementation considerations*. BCTs identified in the interview were considered alongside the least fulfilled NPT core/sub constructs (relational integration, skill set workability, contextual integration and reflexive monitoring). Long-term implementation strategies were designed for relevant BCTs to maximise embeddedness. ***Relational integration*** is work done to build accountability and confidence in an intervention and the people delivering it. ***Role models and champions*** will support this. Long-term implementation will be delivered by formalisation of the delirium champion role including a governance structure, job description and supervision of the role. ***Skills set workability*** is whether the people carrying out the intervention have the correct skills. To ensure embeddedness of skills training the ***educational meetings*** will from part of induction and mandatory training updates. Training sessions will be delivered using a standardised slide deck designed to meet specific training needs. ***Contextual integration*** is whether the correct resources and support are available to carry out the intervention. Electronic ***prompts*, *orientation tools*, *printed guidelines and screening tools*** will be created and monitored according to usual hospice maintenance and governance procedures. Long-term managerial support is available via the hospice’s Evidence-Based Practice Group which is responsible for review of clinical guidelines and audit. ***Reflexive monitoring*** includes the work of determining how effective the intervention is (systemisation). ***Audit and feedback*** will be monitored and ensured through the Evidence-Based Practice group.

#### Staff meeting

Most of the strategies suggested were considered appropriate without modification. However, suggestions included having i) teaching sessions delivered separate to usual mandatory training, several times in one month, to allow all staff an opportunity to attend and ii) those delivering the training attend two ward rounds to role-model behaviours.

## Discussion

### Statement of principle findings

The clinical records review identified delirium screening on admission and non-pharmacological management of delirium as priority areas for improvement. We took a theoretical approach to establishing barriers and facilitators through staff survey and subsequent exploration in staff interviews. Interviews also identified strategies to enhance practice. A theoretical approach was used to consider the implementation of these strategies and they were subsequently refined in a staff meeting. Strategies identified included: provision of role models and champions, educational meetings, skill-mix on staff rota and environmental changes to support systematic screening and non-pharmacological management.

### Strengths and limitations

The study was conducted in a robust manner. To our knowledge, this study is the first to use a theoretical approach to establish barriers and facilitators to delirium care in a hospice and to design strategies to improve these areas of care. Although the work focused on one hospice in England, limiting transferability, it is likely findings are applicable internationally to other palliative care in-patient settings. We acknowledge the limitations of including only eight interviews. However, the ability to synthesise these data with survey was a strength, and interview data saturation was achieved after five interviews–consistent with similarly theoretically underpinned interview studies [20].

### Interpretation within the context of the wider literature

Our study found barriers to assessment of delirium previously identified including poor knowledge of delirium symptoms [23–25], a culture of using other words to describe delirium [23, 25], time and workload pressures, and poor access to guidelines [9, 26]. We identified additional barriers, including staff beliefs they can identify delirium without screening and screening being a low priority task; in particular for patients with hypoactive delirium as they cause no management challenge in a cultural environment favouring ‘quiet and peaceful’ [26]. A person’s behaviour may be controlled by determinants they’re not consciously aware of [27]. A strength of using a theoretical approach to questioning is the potential for increased conscious awareness of these influences which may explain the additional barriers we found.

In line with previous literature, we identified barriers to non-pharmacological management of delirium including staff lacking skills to instigate non-pharmacological management or manage challenging patient behaviours [23–25], stress in managing patients with delirium [23, 26] and having other care priorities [23]. We also identified setting-specific barriers including poor availability and maintenance of non-pharmacological management tools and the ward set-up.

### Implications for policy, practice and research

This is the first reported project using the behaviour change TDF and Normalisation Process Theory to design an intervention in a hospice setting. We have shown a theoretical approach to create a bespoke intervention to improve delirium care in a hospice setting is feasible and have designed a theory-derived, multi-component intervention with supporting implementation strategies. This gives other hospices a starting point for designing their own interventions. We recommend colleagues consider local barriers and facilitators and tailor solutions accordingly. However, from our findings and previous literature describing barriers to guideline-adherent delirium care, it is likely there are barriers in common across different care settings and the strategies we suggest may support others.

This project uses a valid case note review as a tool to collect anonymised delirium care process and outcome data from clinical notes. It demonstrates that it is feasible to collect high quality, representative data in this way for many aspects of delirium prevention, recognition, and management for use in clinical quality improvement initiatives or in research.

The findings of this study have been used to inform the study design and educational materials of a recently published feasibility trial of an implementation strategy to improved delirium care in hospices [28] and, subsequently, for a planned evaluation phase cluster trial of 20 hospices.

## Conclusions

In this study, clinical record review established areas of poor concordance with delirium care. Through a survey, interviews with front line staff, and a meeting with senior clinical staff we sought to understand barriers and facilitators to delirium care and to design strategies to improve concordance. Further testing is needed to see if strategies improve guideline-adherent delirium care and clinical outcomes for hospice patients.
